# Long-Acting Injectable Antipsychotics in Schizophrenia and Comorbid Substance Use Disorder: A Systematic Review of the Literature

**DOI:** 10.3390/brainsci16090908

**Published:** 2026-08-26

**Authors:** Matteo Lupi, Alessandro Carano, Eros Rossi, Maria Carlucci, Angelomarco Barioglio, Marco Giri, Domenico De Berardis, Umberto Volpe, Giovanni Martinotti

**Affiliations:** 1 NHS, Mental Health Department, AST Ascoli Piceno, 63100 Marche, Italy; alessandro.carano@gmail.com (A.C.); eros.rossi@sanita.marche.it (E.R.); mariacarlucci.psy@gmail.com (M.C.); angelomarco.barioglio@sanita.marche.it (A.B.); marco.giri@sanita.marche.it (M.G.); 2 NHS, Mental Health Department, ASL Teramo, 64100 Abruzzo, Italy; domenico.deberardis@aslteramo.it; 3 Unit of Clinical Psychiatry, Department of Clinical Neurosciences/DIMSC, School of Medicine, Polytechnic University of Marche, 60121 Ancona, Italy; prof.umberto.volpe@gmail.com; 4 Department of Neuroscience, Imaging and Clinical Sciences, University “G. d’Annunzio”, 66100 Chieti-Pescara, Italy; giovanni.martinotti@gmail.com

**Keywords:** long-acting injectable antipsychotic, schizophrenia, psychosis, substance use disorder

## Abstract

**Highlights:**

**What are the main findings?**

- Nine studies involving 2062 patients with schizophrenia and comorbid substance use disorder (SUD) were included, with follow-up periods ranging from 6 months to 3 years.
- Several studies reported favorable clinical outcomes with long-acting injectable (LAI) antipsychotics, but the evidence was mixed and did not consistently demonstrate superiority over oral antipsychotics.

**What are the implications of the main findings?**

- LAI antipsychotics may be a valuable treatment option for patients with schizophrenia and comorbid SUD, particularly where adherence to oral medication is a concern.
- The heterogeneity of the available evidence highlights the need for more high-quality, standardized research to better establish the efficacy, effectiveness, and safety of LAIs in this patient population.

**Abstract:**

**Background/Objectives:** The aim of this systematic review is to assess the efficacy, effectiveness, and safety of LAI antipsychotics in patients with schizophrenia and comorbid SUD, providing useful information for clinical decision making and guiding future research in the management of this complex patient population. **Methods:** The search was conducted on 1 July 2026 and produced 87 results in total; after the screening process, 78 articles were excluded based on the predefined eligibility criteria. The full texts of the remaining 9 studies were retrieved and thoroughly evaluated for inclusion in the qualitative synthesis. **Results:** The 9 articles that met the criteria for inclusion included 2062 patients in total with follow-up periods varying between 6 months and 3 years. Several studies reported favorable clinical outcomes associated with LAIs; however, not all studies demonstrated clear superiority of LAIs over oral antipsychotics. **Conclusions:** The present review suggests that LAIs may represent a valuable therapeutic strategy for patients with schizophrenia and comorbid SUD, although the available evidence remains heterogeneous in terms of study design, patient populations, and outcome measures.

## 1. Introduction

Schizophrenia is a serious mental disorder with a lifetime prevalence of approximately 0.5–1% and has a considerable burden on the healthcare system [1]. Schizophrenia is often associated with comorbid substance use disorders (SUDs), with the prevalence of dual disorders ranging from 26% to 31% [2]; substance use disorders are also associated with an early onset of schizophrenia [3].

The relationship between schizophrenia and substance use is complex and bidirectional. On one hand, substance use may precipitate or accelerate the onset of psychosis in genetically or biologically vulnerable individuals. On the other hand, many individuals with schizophrenia develop substance use disorders after illness onset, possibly as a consequence of attempts to alleviate distress, reduce negative symptoms, counteract medication-related adverse effects, or improve social functioning [4]. Shared neurobiological mechanisms involving dopaminergic dysfunction, reward processing, impulsivity, cognitive impairment and genetic susceptibility further support the existence of overlapping pathways between schizophrenia and addiction [5].

Individuals with schizophrenia and comorbid SUDs often experience poor treatment adherence, leading to worse clinical outcomes [6,7].

Both schizophrenia and substance use disorder are associated with cognitive impairment; non-adherence in patients with dual diagnosis is strongly driven by neurocognitive impairments and reduced insight into the illness. Substance abuse exacerbates deficits in working memory and executive functions, making the daily management of oral therapy complex and prone to failure.

In this context, long-acting injectables (LAIs) overcome the barrier posed by cognitive impairment by eliminating the need for daily planning by the patient [5].

The rationale for antipsychotic treatment in patients with schizophrenia and co-occurring SUD is based primarily on the need to control psychotic symptoms while also potentially addressing clinical factors that contribute to substance-related morbidity. Schizophrenia and SUD share several neurobiological and clinical mechanisms, including alterations in dopaminergic reward and salience pathways, which may contribute to both psychotic symptoms and substance-related behaviors. Persistent psychotic symptoms, impaired judgment, craving, cognitive dysfunction, and psychosocial instability may further interfere with treatment adherence and engagement with addiction care. Effective antipsychotic treatment may therefore contribute to greater clinical stability and facilitate participation in psychosocial and substance-use interventions.

Within this context, LAI formulations may offer an additional theoretical advantage by providing sustained antipsychotic exposure and reducing the risk of missed doses. This may be particularly relevant in patients with dual disorders, in whom medication non-adherence, recurrent substance use, unstable social circumstances, and repeated hospitalization are common clinical challenges. However, the use of antipsychotics in this context should not be interpreted as evidence that antipsychotic medications are primary treatments for SUD itself. Rather, their potential role is principally related to the treatment of the co-occurring psychotic disorder, with possible indirect effects on substance-related outcomes through improved symptom control and treatment continuity.

In fact, medication adherence remains one of the major challenges in the long-term management of schizophrenia, particularly among patients with co-occurring SUD [8]. Non-adherence to antipsychotic treatment has consistently been linked to symptom exacerbation, relapse, and increased healthcare utilization [9]. Given these challenges, LAIs antipsychotics have gained increasing attention as a therapeutic strategy to improve treatment continuity and reduce relapse risk. Compared with oral antipsychotics, LAIs provide sustained medication delivery, facilitate monitoring of adherence, and may enhance engagement with mental health services [10]. These potential benefits may be particularly relevant in individuals with dual diagnoses, where treatment discontinuation and substance use frequently complicate clinical management [11].

Schizophrenia patients initiating LAI antipsychotics also incur lower healthcare costs in comparison to patients initiating oral antipsychotics; a recent analysis of patients before and after the initiation of LAI treatment shows a drastic drop in total monthly costs per patient, driven by a sharp decrease in the duration of hospitalizations [12].

Moreover the caregiving burden is significantly higher in cases of dual diagnosis compared to schizophrenia alone. With oral formulations, family members are often compelled to obsessively monitor daily medication intake, triggering stressful and conflict-ridden relational dynamics. Switching to LAIs de-escalates these interpersonal conflicts, relieving the family of the monitoring role and improving the therapeutic atmosphere within the household [13].

However, despite increasing clinical use of LAIs, evidence regarding their effectiveness in patients with schizophrenia and comorbid SUD remains limited. Most randomized controlled trials evaluating antipsychotic treatments have historically excluded patients with active substance use, resulting in a significant gap in the literature [4]. Recent systematic reviews have suggested that LAIs may improve both psychiatric and substance use outcomes in this population, although the available evidence is characterized by methodological heterogeneity, small sample sizes, and limited long-term follow-up [14].

Given the substantial clinical burden associated with schizophrenia and co-occurring SUD, as well as the growing emphasis on personalized and adherence-focused treatment strategies, a comprehensive evaluation of the available evidence is warranted. A better understanding of the benefits and limitations of LAI antipsychotics in this population may help clinicians optimize pharmacological treatment, improve long-term outcomes, reduce healthcare utilization, and support integrated management approaches addressing both psychotic illness and substance use.

Therefore, the aim of this systematic review is to assess the efficacy, effectiveness, and safety of LAI antipsychotics in patients with schizophrenia and comorbid SUD, providing useful information for clinical decision making and guiding future research in the management of this complex patient population.

## 2. Methods

The systematic literature search was conducted in accordance with the Preferred Reporting Items for Systematic Reviews and Meta-Analyses (PRISMA) 2020 statement [15]. The review protocol was not prospectively registered in PROSPERO or any other systematic review registry.

The final electronic search of PubMed was performed on 1 July 2026, using three core conceptual domains: (1) long-acting injectable antipsychotics, (2) schizophrenia or psychotic disorders, and (3) substance use disorder. The initial PubMed search identified 87 records.

To identify additional potentially relevant publications, Scopus, PsycINFO, and Google Scholar were subsequently searched using the same three core conceptual domains. The search strategies were adapted to the specific search syntax, indexing systems, and functionality of each database. In Scopus, the search was conducted using title, abstract, and keyword fields. In PsycINFO, the corresponding concepts were searched using title and abstract fields, with database-specific indexing terminology considered where applicable. Google Scholar was searched using combinations of the three core concepts because of its different search functionality.

Records retrieved from all electronic sources were manually compared during the screening process, and duplicate records were identified and removed manually without the use of dedicated reference-management or screening software. Titles and abstracts were independently screened by two reviewers according to the predefined eligibility criteria. Disagreements were resolved through discussion and consensus.

Studies were eligible if they specifically investigated the use of long-acting injectable antipsychotics in individuals with schizophrenia or a psychotic disorder and co-occurring substance use disorder. Eligible study designs included randomized controlled trials, controlled clinical trials, observational studies, prospective clinical studies, and other clinical studies meeting the predefined eligibility criteria. Studies were excluded if they were review articles, systematic reviews, meta-analyses, editorials, letters, or educational or didactic papers or if they did not specifically investigate long-acting injectable antipsychotics in individuals with schizophrenia or a psychotic disorder and co-occurring substance use disorder. Studies that did not provide sufficient information relevant to the objectives of the present review were also excluded. Only studies published in English and with an available abstract were considered.

**Figure 1:**
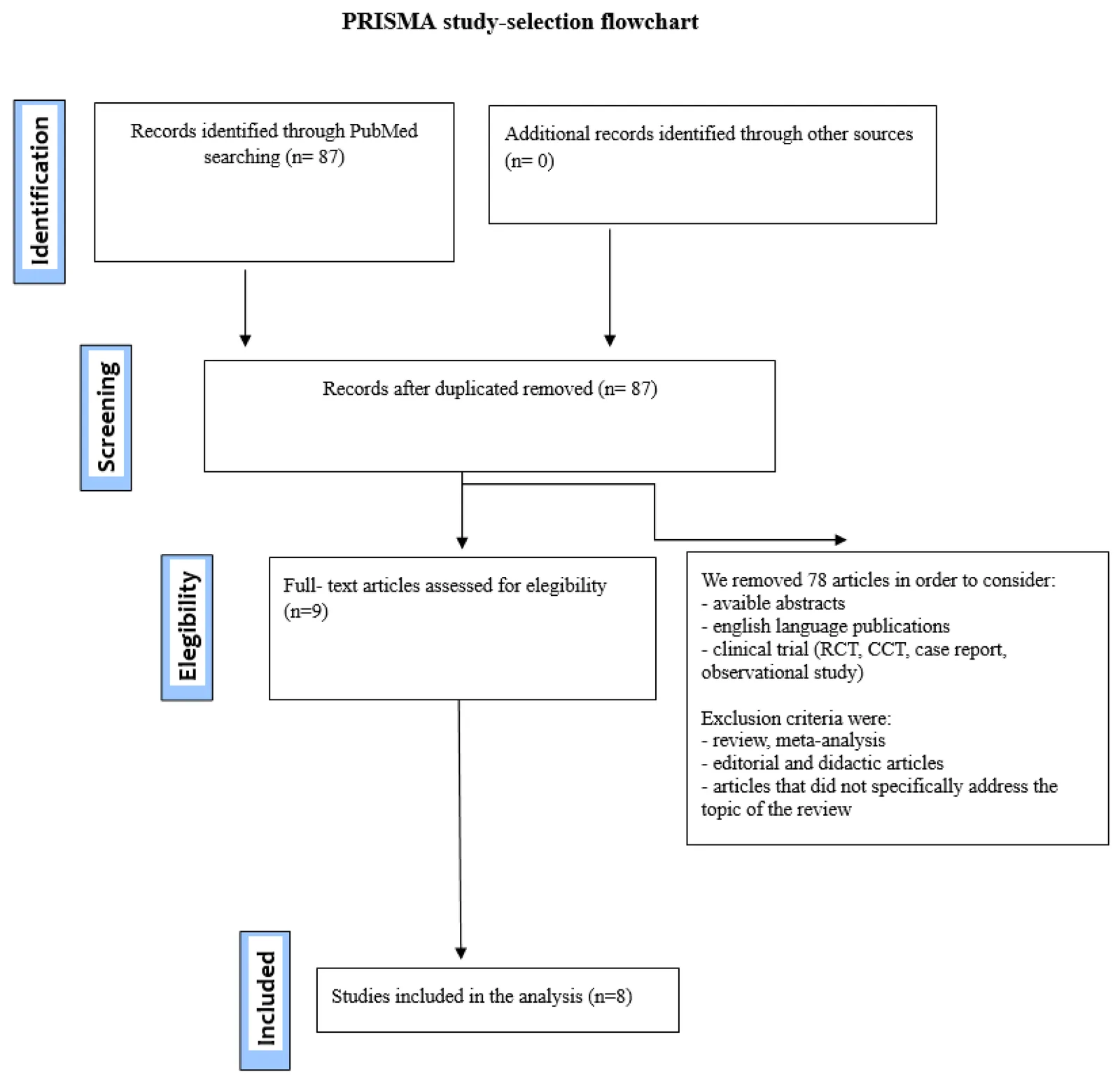
PRISMA study selection flowchart.

Following title and abstract screening, 78 records were excluded according to the predefined eligibility criteria. The full texts of the remaining nine articles were retrieved and assessed for eligibility. All nine studies met the inclusion criteria and were included in the qualitative synthesis. Data from the included studies were examined to summarize the available evidence regarding the efficacy, safety, tolerability, adherence, and other clinically relevant outcomes associated with long-acting injectable antipsychotic treatment in this patient population. The study-selection process is summarized in Figure 1.

### 2.1. Data Extraction

Data extraction was performed independently by two reviewers using a predefined data-extraction approach. The reviewers assessed study characteristics, including study design, country, sample size, patient population, type of substance use disorder, LAI antipsychotic administered, comparator where applicable, treatment duration, clinical outcomes, and adverse events. The two reviewers reached consensus on all extracted information.

### 2.2. Risk of Bias and Methodological Quality Assessment

The methodological quality and risk of bias of the included studies were assessed independently by two reviewers using validated tools appropriate to the design of each study.

For randomized controlled trials, risk of bias was assessed using the Cochrane Risk of Bias 2 (RoB 2) tool. For the observational comparative studies, risk of bias was assessed using the Risk Of Bias In Non-randomized Studies of Interventions (ROBINS-I) tool. The prospective uncontrolled study was assessed using the NIH Quality Assessment Tool for Before–After (Pre–Post) Studies With No Control Group (data is summarized in Table 1).

The two reviewers independently performed the risk-of-bias and methodological quality assessments and reached consensus on all assessments.

## 3. Results

The 9 articles that met the criteria for inclusion included 2062 patients in total; sample sizes ranged from 30 to 471 participants, with follow-up periods varying between 6 months and 3 years.

The included studies were heterogeneous with respect to design, encompassing randomized controlled trials (RCTs), open-label comparative studies, prospective uncontrolled investigations, retrospective observational analyses, and post hoc exploratory analyses of larger clinical trials. Similarly, substantial variability was observed in the antipsychotic formulations investigated, including LAI risperidone, paliperidone palmitate, aripiprazole monohydrate, haloperidol decanoate, and zuclopenthixol depot.

Mortazavi et al. (2026) [6] performed a multicenter, randomized, open-label trial across several European healthcare settings. This secondary analysis included 471 patients with early-phase schizophrenia, of whom 143 had comorbid SUD and 328 had no history of SUD. Participants were randomized to receive either a second-generation LAI antipsychotic or an oral second-generation antipsychotic. Among patients with comorbid SUD, treatment with LAIs was associated with a 36% reduction in the risk of all-cause treatment discontinuation compared with oral antipsychotics. No significant advantage of LAIs was observed among patients without SUD, suggesting that the benefit of injectable formulations may be particularly relevant in dual-diagnosis populations [6].

A randomized clinical trial conducted between 2005 and 2008 evaluated the comparative effectiveness of oral and LAI risperidone in 95 patients meeting DSM-IV-TR diagnostic criteria for schizophrenia and alcohol use disorder (AUD). Participants were randomly allocated to receive either oral risperidone or long-acting injectable risperidone for six months, with outcomes including alcohol consumption, psychiatric symptoms, medication adherence, and treatment retention. Although patients in both treatment groups continued to consume alcohol during follow-up, individuals receiving LAI risperidone demonstrated greater treatment adherence and superior overall clinical stability than those treated with the oral formulation. Improvements were observed in psychiatric symptom control despite the persistence of alcohol use, suggesting that LAI administration may partially mitigate the detrimental effects of ongoing substance use on treatment continuity. While complete abstinence was not achieved, the investigators concluded that injectable risperidone represented a clinically preferable option for patients with schizophrenia and comorbid AUD, particularly in those at elevated risk of medication non-adherence [16].

Rubio et al. (2006) [17] performed one of the earliest randomized controlled studies specifically designed to investigate LAI antipsychotics in patients with dual diagnosis. The study enrolled 115 individuals with schizophrenia and co-occurring substance abuse disorders who were randomly assigned to receive either long-acting injectable risperidone (n = 57) or zuclopenthixol depot (n = 58) over a six-month follow-up period. Both treatment groups demonstrated improvements in psychotic symptoms; however, patients receiving LAI risperidone experienced significantly greater reductions in substance use severity, craving, and overall psychopathology than those treated with the first-generation depot formulation. Moreover, improvements in global functioning and treatment adherence were more pronounced among patients treated with risperidone. These findings suggested that second-generation LAIs may offer advantages over first-generation depot antipsychotics in patients with dual diagnosis, potentially owing to their more favorable tolerability profile, lower propensity for extrapyramidal adverse effects, and greater efficacy against negative and affective symptoms, all of which may contribute to improved engagement with treatment [17].

An exploratory post hoc analysis evaluated the effectiveness of once-monthly paliperidone palmitate compared with daily oral antipsychotics in patients with schizophrenia and a history of incarceration, a population recognized as being at particularly high risk for treatment discontinuation and recurrent hospitalization. Participants were stratified according to the presence or absence of substance abuse to determine whether substance use modified treatment response. Across both subgroups, paliperidone palmitate significantly prolonged the time to treatment failure compared with oral antipsychotics, confirming the overall superiority of LAI therapy in maintaining clinical stability. Nevertheless, although the injectable formulation remained beneficial among patients with substance abuse, the magnitude of its effect was attenuated compared with individuals without substance use. This observation suggests that while LAIs improve adherence and reduce relapse risk, ongoing substance misuse may continue to negatively influence clinical outcomes through mechanisms extending beyond medication adherence alone, including persistent neurobiological vulnerability, psychosocial instability, and reduced engagement with non-pharmacological interventions [18].

Sajatovic et al. (2013) [19] conducted a prospective uncontrolled trial of customized adherence enhancement (CAE) plus LAI using haloperidol decanoate in 30 homeless or recently homeless individuals with DSM-IV-defined schizophrenia or schizoaffective disorder; most (97%) had past or current substance abuse and had been incarcerated. Participants received monthly CAE and LAI (CAE-L) for 6 months. While interpretation of findings must be tempered by the methodological limitations, CAE-L appears to be associated with improved adherence, symptoms, and functioning in homeless or recently homeless individuals with schizophrenia or schizoaffective disorder [19].

In a paper by Leatherman et al. (2014) [20], patients with schizophrenia or schizoaffective disorder who had been hospitalized within the past 2 years or judged to be at risk for hospitalization because of increasing psychiatric service use were randomly assigned to LAI risperidone 12.5 to 50 mg per injection biweekly or to the psychiatrist’s choice of oral antipsychotics and followed for up to 2 years. Cox’s regression and mixed effects models were used to assess difference in treatment effect within 12 subgroups defined by hospitalization at study entry, substance abuse, race, symptom severity, quality of life, body mass index, age, race or sex, or reported medication compliance. LAI risperidone showed no superiority to psychiatrist’s choice of oral treatment in most clinically defined subgroups, although the white patients benefited more than the other groups in terms of substance abuse outcomes [20].

An observational retrospective study conducted under routine clinical practice conditions evaluated the impact of LAI treatment on hospitalization outcomes in a cohort of 170 patients with schizophrenia-spectrum disorders. The investigators compared hospitalization rates and total hospitalization days before and after initiation of LAI antipsychotic therapy and also examined differences between patients maintained on injectable formulations and those receiving oral antipsychotics. Following initiation of LAIs, the incidence of psychiatric hospitalization decreased by approximately 89%, accompanied by a substantial reduction in cumulative hospitalization days. These findings translated into a marked decrease in healthcare resource utilization, supporting the potential economic benefits of sustained treatment adherence achieved through LAI administration. Importantly, the presence of comorbid SUD independently increased hospitalization rates by approximately 123%, confirming SUD as one of the strongest predictors of poor clinical outcomes. The authors suggested that future prospective studies should determine whether LAIs should be considered earlier in the course of illness, particularly among patients experiencing first-episode psychosis or those presenting with co-occurring substance use disorders, who appear to be at particularly high risk of relapse and repeated hospitalization [21].

Cuomo et al. (2018) [22] directly compared the effectiveness of two second-generation LAI antipsychotics in patients with psychotic disorders and comorbid SUD. A total of 101 initially hospitalized patients were randomly assigned to receive either once-monthly intramuscular aripiprazole monohydrate (400 mg; n = 50) or once-monthly paliperidone palmitate (100 mg; n = 51). Following stabilization during hospitalization, patients were discharged and prospectively followed for twelve months. Clinical status, substance craving, psychopathology, and quality of life were systematically assessed throughout the study period. Both treatment groups demonstrated significant improvements in psychiatric symptoms, global functioning, and quality of life, together with meaningful reductions in substance craving over the one-year follow-up. However, patients treated with aripiprazole monohydrate experienced significantly greater improvements in craving intensity and quality-of-life measures than those receiving paliperidone palmitate, while both formulations showed comparable efficacy in controlling psychotic symptoms. These findings suggest that although second-generation LAIs generally appear beneficial in dual-diagnosis populations, pharmacological differences between individual agents may influence specific clinical domains, particularly those related to reward processing, motivational functioning, and substance craving [22].

Finally, Abdel-Baki et al. (2020) [23] investigated the effectiveness of LAIs as first-line treatment in 237 patients with first-episode psychosis (FEP) and comorbid SUD followed for three years. Thirty-one patients initially received an LAI antipsychotic, whereas 206 received oral antipsychotics. Despite presenting with poorer baseline prognostic characteristics, including a higher prevalence of homelessness, patients treated initially with LAIs experienced lower relapse rates (67.7% vs. 76.7%), significantly longer relapse-free survival (694 vs. 447 days; *p* = 0.008), and a trend toward fewer rehospitalizations compared with those receiving oral antipsychotics. Moreover, over 40% of patients initially treated with oral antipsychotics were subsequently switched to LAIs because of poor clinical outcomes, further supporting the potential role of LAIs in this high-risk population [23] (data are summarized in Table 2).

## 4. Discussion

The present review suggests that LAIs may represent a valuable therapeutic strategy for patients with schizophrenia and comorbid SUD, although the available evidence remains heterogeneous in terms of study design, patient populations, and outcome measures.

One of the most consistent findings across the included studies is the beneficial effect of LAIs on treatment adherence and continuity. The recent randomized trial by Mortazavi et al. (2026) [6] demonstrated that LAIs reduced the risk of all-cause treatment discontinuation by 36% in patients with early-phase schizophrenia and comorbid SUD, whereas no significant advantage was observed in patients without SUD. This finding supports the hypothesis that patients with dual diagnosis may derive greater benefit from LAIs because they are particularly vulnerable to poor adherence, medication discontinuation, and relapse [6].

Several studies also reported favorable clinical outcomes associated with LAIs. Randomized studies comparing LAI risperidone with oral risperidone or zuclopenthixol depot found improvements not only in psychotic symptoms but also in substance use outcomes, suggesting that enhanced medication adherence may indirectly contribute to better control of addictive behaviors [17]. Similarly, Cuomo et al. demonstrated that both aripiprazole monohydrate and paliperidone palmitate improved psychopathology, quality of life, and craving, with aripiprazole showing greater benefits in craving reduction and quality-of-life measures after one year of treatment [22].

The evidence regarding relapse prevention and hospitalization is also generally favorable. Observational studies consistently reported reductions in hospitalization rates following LAI initiation, while Abdel-Baki et al. found longer relapse-free survival and a trend toward fewer rehospitalizations among first-episode psychosis patients with SUD receiving LAIs, despite their poorer baseline prognostic characteristics [23]. These findings suggest that the pharmacological advantages of continuous drug delivery may be particularly relevant in individuals at high risk of treatment interruption.

However, not all studies demonstrated clear superiority of LAIs over oral antipsychotics. Leatherman et al. did not observe significant differences across most clinically defined subgroups, while the exploratory analysis of paliperidone palmitate showed that although treatment failure was reduced compared with oral antipsychotics, the magnitude of benefit was attenuated among patients with substance abuse [20]. These discrepant findings likely reflect differences in study populations, specific LAI formulations, severity and type of substance use, duration of follow-up, and methodological approaches.

An important consideration emerging from the available evidence is that pharmacological treatment alone may not be sufficient for this particularly vulnerable population. The study by Sajatovic et al. showed that combining LAIs with Customized Adherence Enhancement (CAE) was associated with improvements in adherence, symptoms, and functioning among homeless individuals with schizophrenia, nearly all of whom had comorbid substance use [19]. This supports an integrated treatment model in which LAIs are combined with psychosocial interventions, addiction treatment, and case management to maximize clinical outcomes.

However, the evidence remains heterogeneous, and not all studies have demonstrated a clear superiority of LAIs over oral antipsychotics. This variability may be explained by differences in study designs, patient characteristics, types of substances of abuse, and the antipsychotic agents investigated [24].

The present review also highlights several methodological limitations of the included studies. The investigations employed a range of LAI formulations with different dosages and dosing intervals, making cross-study comparisons difficult. In addition, a wide variety of substances of abuse were included, with many of the larger studies enrolling participants with concurrent polysubstance use, further increasing heterogeneity. Follow-up duration also varied considerably, ranging from a few weeks to 12 months, whereas robust conclusions regarding the efficacy and safety of LAIs require long-term follow-up data [25,26].

## 5. Conclusions

The findings from randomized studies should be distinguished from those derived from observational and uncontrolled investigations. The randomized trials provide the most robust evidence regarding comparative treatment effects, although they also differed substantially in design and objectives. For example, Mortazavi et al. reported a lower risk of all-cause treatment discontinuation with LAIs compared with oral antipsychotics among patients with schizophrenia and comorbid SUD, whereas Leatherman et al. did not identify a consistent superiority of LAI risperidone over psychiatrist-selected oral antipsychotic treatment across the clinical subgroups examined. Similarly, Rubio et al. and Cuomo et al. reported favorable outcomes associated with specific LAI formulations, although these studies compared different active treatments and evaluated different clinical outcomes. These findings therefore suggest potential benefits of LAI treatment in selected clinical domains rather than establishing a uniform superiority of LAIs over oral antipsychotics.

The observational studies provide complementary but less definitive evidence. Martín-Muñoz et al. reported a substantial reduction in hospitalization rates following LAI initiation, while Abdel-Baki et al. observed longer relapse-free survival among patients with first-episode psychosis and comorbid SUD who initially received LAIs. However, these findings must be interpreted in light of the non-randomized nature of these studies and the possibility of confounding by baseline clinical characteristics, treatment selection, adherence, and other factors. The prospective uncontrolled study by Sajatovic et al. further suggested improvements in adherence, symptoms, and functioning following the combination of customized adherence enhancement and LAI treatment; however, the absence of a control group substantially limits causal interpretation.

The available evidence suggests that long-acting injectable antipsychotics may be a useful treatment option for selected patients with schizophrenia or psychosis and co-occurring substance use disorder. Across the included studies, LAIs were associated with favorable outcomes in treatment continuity and adherence, while some studies also reported improvements in psychiatric symptoms, hospitalization, relapse, craving, or quality of life. However, these findings should be interpreted cautiously because the evidence base is limited and heterogeneous. Randomized controlled trials, observational studies, and uncontrolled investigations provide different levels of evidence, and their findings cannot be considered equivalent. Furthermore, the small number of studies, differences in patient populations and substance-use profiles, variation in LAI formulations and comparators, and the absence of a quantitative synthesis limit the strength and generalizability of the conclusions. Therefore, the current evidence supports considering LAI antipsychotics as an individualized treatment option, particularly for patients with previous difficulties with adherence or treatment continuity, but does not support recommending their routine early use in all patients with dual disorders. Further high-quality randomized studies are required to establish their comparative effectiveness and to identify the clinical characteristics of patients most likely to benefit.

## Figures and Tables

**Table 1:** Risk of bias and methodological quality of the included studies.

	Study Design	Assessment Tool	Risk of Bias/Methodological Quality	Main Methodological Considerations
Mortazavi et al. 2026 [6]	Randomized, open-label trial	Cochrane RoB 2	Some concerns	Randomized design provides relatively strong evidence; however, the analysis of patients with SUD was a secondary/subgroup analysis and treatment allocation was open-label.
Green et al. 2015 [16]	Randomized Controlled Trial	Cochrane RoB 2	Some concerns	Randomized comparative design; open-label treatment may introduce performance and outcome-assessment bias, particularly for subjective outcomes.
Rubio et al. 2006 [17]	Open, randomized, controlled trial	Cochrane RoB 2	Some concerns	Randomized controlled design; open-label nature and potential limitations in blinding may affect subjective clinical and substance-use outcomes.
Lynn Starr et al. 2018 [18]	Prospective, randomized, open-label, event-monitoring board-blinded, active-controlled, multicenter	Cochrane RoB 2	Some concerns	Underlying randomized trial provides stronger evidence, but the present analysis was exploratory/post hoc and focused on a subgroup with substance use.
Sajatovic et al. 2013 [19]	Prospective uncontrolled trial	NIH Quality Assessment Tool for Before–After (Pre–Post) Studies With No Control Group	Moderate/limited quality	Absence of a control group limits causal inference; small sample size and highly selected vulnerable population also limit generalizability.
Leatherman et al. 2014 [20]	Randomized Controlled Trial	Cochrane RoB 2	Some concerns	Randomized comparative design; subgroup analyses and multiple clinical subgroups may limit the certainty of specific subgroup findings
Martín-Muñoz et al. 2023 [21]	Observational, retrospective study	ROBINS-I	Moderate-to-serious risk of bias	Non-randomized treatment allocation, potential confounding by indication, retrospective design, and possible differences between treatment groups.
Cuomo et al. 2018 [22]	Randomized trial	Cochrane RoB 2	Some concerns	Randomized comparative design; open-label treatment and subjective clinical outcomes may introduce potential bias.
Abdel-Baki et al. 2020 [23]	Naturalistic, longitudinal, prospective and retrospective study	ROBINS-I	Moderate-to-serious risk of bias	Non-randomized treatment allocation and important baseline differences between LAI and oral-treatment groups may result in confounding and selection bias

**Table 2:** Abbreviations: LAI, Long-acting Injectable; EULAST, European Long-Acting Antipsychotics in Schizophrenia Trial; SUD, Substance Use Disorder.

	Country	Sample Size	Formulations of LAI	Substance Use Disorder	Major Findings
Mortazavi et al. 2026 [6]	EULAST; 15 European countries and Israel	471	LAI antipsychotics second-generation	Alcohol and Substance Use	Among 143 individuals with schizophrenia and SUD, LAI treatment was associated with a 36% lower risk of all-cause discontinuation compared with oral antipsychotics
Green et al. 2015 [16]	USA	95	LAI risperidone	Alcohol Use Disorder	The data suggested that injectable risperidone may be a better choice than the oral form for these dual diagnosis patients
Rubio et al. 2006 [17]	Spain	115	LAI risperidone Zuclopenthixol-depot	Alcohol and Substance Use	LAI risperidone was more effective than zuclopenthixol-depot in improving substance abuse and schizophrenia symptoms in subjects with dual diagnosis
Lynn Starr et al. 2018 [18]	USA	450	Once-monthly paliperidone palmitate	Substance or alcohol misuse	The observed beneficial effect of paliperidone palmitate was attenuated in the substance-abuse cohort
Sajatovic et al. 2013 [19]	USA	30	Haloperidol decanoate	Substance abuse	Customized Adherence Enhancement plus LAI appears to be associated with improved adherence, symptoms, and functioning
Leatherman et al. 2014 [20]	USA	369	LAI risperidone	Alcohol and drug use	LAI risperidone showed no superiority to psychiatrist’s choice of oral treatment in most clinically defined subgroups, although the white patients benefited more than the other groups in terms of substance abuse outcomes
Martín-Muñoz et al. 2023 [21]	Spain	170	LAIs (aripiprazole and paliperidone/risperidone)	Alcohol and Substance Use	There was an 89% reduction in the incidence of hospitalizations after treatment with LAIs; the presence of a substance use disorder significantly increased the rate of hospitalizations by 123%.
Cuomo et al. 2018 [22]	Italy	125	LAI aripiprazole Once-monthly LAI paliperidone palmitate	Alcohol and Substance Use	In conclusion, 1-year AM and PP was followed by improved clinical status and QoL and reduced substance craving in a population with psychosis and SUD comorbidity.
Abdel-Baki et al. 2020 [23]	Canada	237	LAI antipsychotics	Alcohol and Substance Use	The LAI group presented a lower relapse rate, higher relapse-free survival time, trends for reduced rehospitalization rates and greater hospitalization-free survival time.

## Data Availability

The datasets generated during and/or analyzed during the current study are not publicly available but are available from the corresponding author.
